# Oral gavage delivery of *Cornus officinalis* extract delays type 1 diabetes onset and hyperglycemia in non‐obese diabetic (NOD) mice

**DOI:** 10.1002/2211-5463.13758

**Published:** 2024-01-09

**Authors:** Justin D. Fletcher, Grace E. Olsson, Y. Clare Zhang, Brant R. Burkhardt

**Affiliations:** ^1^ Department of Molecular Biosciences University of South Florida Tampa FL USA; ^2^ Practice of Oriental Medicine Tucson AZ USA

**Keywords:** *Cornus officinalis*, C‐peptide, glucose, non‐obese diabetic mouse, type 1 diabetes

## Abstract

Type 1 diabetes (T1D) is an autoimmune disease initiated by genetic predisposition and environmental influences, which result in the specific destruction of insulin‐producing pancreatic β‐cells. Currently, there are over 1.6 million cases of T1D in the United States with a worldwide incidence rate that has been increasing since 1990. Here, we examined the effect of *Cornus officinalis* (CO), a well‐known ethnopharmacological agent, on a T1D model of the non‐obese diabetic (NOD) mouse. A measured dose of CO extract was delivered into 10‐week‐old NOD mice by oral gavage for 15 weeks. T1D incidence and hyperglycemia were significantly lower in the CO‐treated group as compared to the water gavage (WT) and a no handling or treatment control group (NHT) following treatment. T1D onset per group was 30%, 60% and 86% for the CO, WT and NHT groups, respectively. Circulating C‐peptide was higher, and pancreatic insulitis was decreased in non‐T1D CO‐treated mice. Our findings suggest that CO may have therapeutic potential as both a safe and effective interventional agent to slow early stage T1D progression.

## Graphical abstract

There is an urgent need for interventional therapies to delay or prevent the onset of type 1 diabetes (T1D). *Cornus officinalis* (CO) is an ethnopharmacological agent that may provide a promising source for potential therapeutics for this purpose. This study has demonstrated that CO can inhibit T1D onset and hyperglycemia in the non‐obese diabetic mouse.

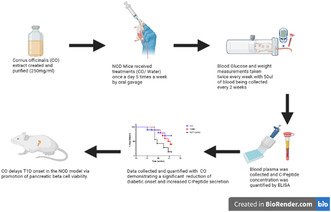

**Abbreviations**
CO: *Cornus officinalis*
CXCL10: C‐X‐C motif chemokine ligand 10
GAD: glutamic acid decarboxylase
GADA: glutamic acid decarboxylase autoantibody
HO‐1: heme oxygenase‐1
IA‐2A: insulinoma‐associated antigen‐2 autoantibodies
IAA: insulin autoantibodies
IFN‐γ: interferon‐gamma
IL‐1β: interleukin 1‐beta
Keap1: Kelch‐like ECH‐associated protein 1
NHT: no handling treatment control
NO: nitric oxide
NOD: non‐obese diabetic mouse
Nrf2: nuclear factor erythroid 2‐related factor 2
ROS: reactive oxygen species
SOD2: superoxide dismutase‐2
T1D: type 1 diabetes
TNF‐α: tumor necrosis factor‐alpha
WT: water treatment group
ZnT8A: zinc transporter 8 autoantibodies

Type 1 diabetes (T1D) is an autoimmune disease initiated by genetic predisposition and environmental influences resulting in the specific destruction of the insulin‐producing pancreatic β‐cells. Currently, there are over 1.6 million cases of T1D in the United States with a worldwide incidence rate that has been increasing since 1990 [1]. Unfortunately, standard care for T1D has not changed since the discovery of insulin 100 years ago and mostly revolves around treating T1D with exogenous insulin following clinical presentation of hyperglycemia which is considered a fasting blood glucose concentration above 126 mg·dL^−1^ or random blood glucose concentration above 200 mg·dL^−1^ [2]. The pathogenesis of T1D typically precedes clinical onset of symptoms due to the progressive destruction of the pancreatic β‐cells by autoimmunity. Approximately 90% of the pancreatic β‐cell mass has been lost at the time of eventual clinical presentation which can take months to years thereby providing a potential interventional opportunity to halt the autoimmune process or salvage remaining functional pancreatic β‐cells.

Ninety percent of T1D patients develop autoantibodies against pancreatic β‐cell‐associated antigens, such as insulin autoantibodies (IAA), GAD autoantibodies (GADA), insulinoma‐associated antigen‐2 autoantibodies (IA‐2A), and zinc transporter 8 autoantibodies (ZnT8A) [1]. Research has shown that individuals who express two or more of autoreactive antibodies have an 84% chance of developing T1D by the age of 18 however, it may take up to 15 years between the detection of the autoreactive antibodies and the onset of T1D symptoms [3]. Even in adult onset T1D which modern research has shown has a lower number of autoreactive antibodies, GAD autoantibodies still remain prevalent [4]. For this reason, between the detection of autoreactive antibodies and the clinical onset of T1D there is a generous interventional window for treatment. Currently, there is only one approved T1D interventional therapy known as Teplizumab. Unfortunately, Teplizumab only had a median delay in the diagnosis of T1D of about 2 years [5] and with an expected high cost of $194 000 for treatment the search for cheaper, effective, and safe T1D interventional therapies remains warranted.

Ethnopharmacological approaches provide a tremendous resource for the discovery of new therapeutics and have resulted in the discovery of important diabetes medications such as the type 2 diabetes medication, metformin [6]. One promising source of new therapeutics is from the frequently utilized herbal application of *Cornus officinalis* (CO). CO extract contains a wide array of biologically active constituents that provide protective cellular properties [7, 8, 9]. Some of the biological actions of CO include protection from oxidative stress [10] and our current research has also shown that CO can protect a pancreatic cell line from cytokine‐induced cell death as well as increase viability and proliferation of a β‐cell pancreatic cell line [11]. Furthermore, CO can induce the activation of the Kelch‐like ECH‐associated protein 1 (Keap1)/nuclear factor erythroid 2‐related factor 2 (Nrf2) pathway which controls the upregulation of crucial antioxidant enzymes such as Heme‐oxidase 1 (HO‐1) and Superoxide dismutase‐2 (SOD2) [12].

In T1D, damage mediated by oxidative stress is a major contributor to the pathogenesis of T1D as well as the diabetic complications that come as a result [13]. This is because oxidative stress plays a major role in T1D induced β‐cell death. The autoimmune response in T1D results in the infiltration of macrophages, dendritic and T cells into the pancreatic islets. These immune cells release several proinflammatory cytokines including but not limited to interferon‐gamma (IFN)‐γ, tumor necrosis factor‐alpha (TNF‐α) and interleukin 1‐beta (IL‐1β). Furthermore, additional harmful reactive molecules such as nitric oxide (NO) and various other reactive oxygen species (ROS) are secreted. This detrimental combination of cytokines and ROS has been shown to play a major role in the apoptosis of the β‐cells in both human and animal models with T1D [14]. This increase in ROS results in β‐cell dysfunction and decreased insulin secretion [15] and when combined with the fact that β‐cells have lower expression of antioxidant enzymes when compared to other tissues [16], β‐cells are extremely susceptible to apoptosis induced by oxidative stress. For this reason, the activation of critical antioxidant pathways such as the Keap1/Nrf2 pathway have been show to repress the onset of T1D by protecting the β‐cells from the oxidative stress caused by inflammation [17].

Since we have shown that CO extracts can induce the Keap1/Nrf2 pathway and promote the stimulation of important antioxidant response genes such as HO‐1 and SOD 1/2 in a pancreatic β‐cell line [12], we wanted to extrapolate these prior findings into *in vivo* models of T1D to initially determine potential clinical efficacy. We hypothesize that CO may be able to delay onset of T1D in the non‐obese diabetic (NOD) mouse and may prove useful in discovering new interventional therapeutics for T1D. To determine the therapeutic effect of CO in T1D, we utilized the well‐established NOD mouse model. NOD mice are prone to developing spontaneous T1D with 90% of female and 52% of male NOD mice developing T1D by 30 weeks of age [18]. A measured dose of CO extract was delivered into NOD mice via oral gavage and efficacy was determined based on measurement of glucose levels, diabetic onset, pancreatic insulitis and C‐peptide.

## Materials and methods

### Animal husbandry

NOD/ShiLtJ (Strain #: 001976) 8‐week‐old female mice were purchased from Jackson Laboratories (Bar Harbor, ME, USA) and housed at 25 °C and 60% humidity and maintained on a 12‐h light/dark cycle in the USF College of Medicine vivarium. Prior to experimentation, female NOD mice were allowed to acclimate to the housing facility for 2 weeks. Mice had unlimited access to laboratory chow (Research diets, 24% protein, 41% carbohydrates, and 24% fat) except for a 4 h. fast prior to glucose measurement whereby food but not water was removed. Husbandry and experimental procedures were reviewed and approved by the USF Institutional Animal Care and Use Committee under Protocols #IS00009521 and #IS00008962.

### Preparation of *Cornus officinalis*

Therapeutic grade *C. officinalis* (CO) (Evergreen Herbs, Shan Zhu Yu, City of Industry, CA, USA) was kindly provided by C. Zhang (Oriental Medicine, Tuscon, AZ, USA) and prepared by water extraction as previously described [12, 19]. A certificate of analysis provided by the supplier has indicated that the extract has been validated as sourced from the fruit of *C. officinalis* and is free of microbiological or heavy metal contamination. The solution was then centrifuged to pellet any insoluble material then filter sterilized with a 0.2‐μm syringe filter. The extracts were then stored in 50 mL aliquots at 4 °C until use at a concentration of 250 mg·mL^−1^ that were subsequently delivered by oral gavage.

### Oral gavage delivery of CO into NOD mice

Twenty‐seven female NOD mice were randomly split into 3 treatment groups, 10 mice receiving a CO treatment at 250 mg·mL^−1^ at a volume of 200 μL once per day 5× per week during weekdays with no treatments over weekend, 10 mice receiving 200 μL of deionized water once per day 5 times per week, and the third group of 7 mice receiving no treatment or handling. Oral gavage treatment was started when mice were at 10 weeks of age. We chose this timepoint to initiate CO treatment because this is when the NOD mouse model typically begins development of inflammation (8–11 weeks) within the pancreas and precedes onset of cytotoxicity and eventual overt clinical diabetes [20]. It is at this stage of T1D in the NOD that we would parallel with that in human T1D for use of an interventional agent. Oral gavage administration of the NOD mice continued for 15 consecutive weeks. Treatment was stopped when mice were 25 weeks old with final specimen collection and termination of experiment with mice aged 26 weeks.

### Glucose and C‐peptide measurements

Fasting blood glucose (FBG) readings were taken every 4 days via tail vein method using the Contour Next glucometer (Ascensia Diabetes Care Inc., East Mississauga, ON, Canada). Body weight was measured every 7 days. One FBG measurement above 130 mg·dL^−1^ was considered a hyperglycemic event and two consecutive FBG separated by at least 72 h above 250 mg·dL^−1^ was classified as diabetic onset upon which mice were subsequently humanely euthanized. Fifty microliter of blood was collected from each mouse via tail vein every 2 weeks using a heparin‐coated Microvette CB 300 LH (Thermo Fisher Scientific, Waltham, MA, USA; Catalog No. NC9046728, ref. 16.443.100). The Microvette CB 300 LH tubes were then centrifuged for 10 min at 1000 ***g*** for plasma collection. Plasma was then collected and aliquoted into Eppendorf tubes. Five microliter of plasma was then used to test for C‐peptide concentrations were measured from collected plasma every 2 weeks during NOD animal study using the Crystal Chem C‐peptide ELISA kit (Elk Grove Village, IL, USA; cat. 90050) following the manufacturer's instructions.

### Immunohistochemistry/insulitis scoring

After the final blood glucose and weight readings at 26 weeks of age, the remaining mice were humanely euthanized by CO_2_ asphyxiation and then exsanguinated via cardiac puncture with subsequent collection of pancreata. The isolated pancreata were fixed in formalin for 24 h, then formalin was removed, and pancreata were immersed in PBS and shipped to the University of Florida Molecular Pathology Core. Fractions were sectioned and stained with hematoxylin and eosin (HE) to determine degree of insulitis per islet in mice treated with CO compared to control with light microscopy using Leica Aperio eSlide manager as previously described. Insulitis was scored from stage 0 to 4. Stage 0 was considered when there was no presence of insulitis with typical islet architecture. Stage 1 was considered when there was perinsulitis but no evidence of immune infiltrate into the islet itself. Stage 2 was considered with immune infiltrate in < 50% of the total islet. Stage 3 was considered when immune infiltrate was > 50% of the total islet. Stage 4 was considered when there was pronounced islet atrophy or 100% immune infiltration.

### Insulitis immunophenotyping

Immunohistochemistry (IHC) was performed to determine the immunological composition of the observed insulitis and was performed as previously described [21]. In brief, paraffin processing was performed on blocks examined during the insulitis scoring for the various treatment groups. The staining series was designed to immunophenotype the insulitis for the cellular populations of total leukocytes [CD45], T‐cells [CD3], and B‐cells [CD45R].

### Multiplexing analysis measuring TNF‐α and CXCL10

Cytokine levels were determined using a multiplexed bead immunoassay and measured with a Luminex MAGPIX instrument (Luminex, Austin, TX, USA) as previously detailed [22, 23]. In brief, TNF‐α and CXCL10 were measured using magnetic bead assay (Mouse XL Cytokine Luminex Performance Premixed Kit; Bio‐Techne, Minneapolis, MN, USA) following manufacturer's instructions. Each assay was analyzed on the Luminex MAGPIX instrument to measure inflammatory concentrations followed by a 5‐parameter logistic curve‐fitting method from a standard curve of each respective analyte. Plasma samples were normalized by equivalent volume of commercially supplied dilution buffer and examined in duplicate per each assay run. Concentration was calculated by the statlia Immunoassay Analysis software (Brendan Bioanalytics, Carlsbad, CA, USA).

### Statistical significance

Statistical significance was determined by log‐rank test for trends and mixed effect analysis (graphpad prism version 5.01, GraphPad Software, Boston, MA, USA). *P* value < 0.05 was considered significant.

## Results

### Experimental design

To determine the biological impact of CO on T1D onset and hyperglycemia in the NOD model within a measured dosage, we established an experimental design that consisted of 3 treatment groups of: CO‐treated (CO, *n* = 10), water‐treated (WT, *n* = 10), and a no handling or treatment control group (NHT, *n* = 7). CO and water were administered once per day for five consecutive days per week for 15 weeks into female NOD mice beginning at 10 weeks of age. The NHT group was included because our earlier experiments (unpublished observation) indicated that the procedure of oral gavaging delayed the onset of T1D in the NOD model. Therefore, we included the NHT group that was not handled or gavaged during course of the study to account for the observed handling/procedure induced T1D delay in the NOD model.

### Chronic oral gavage delivery of CO did not impair weight gain in NOD mice

**Fig. 1:**
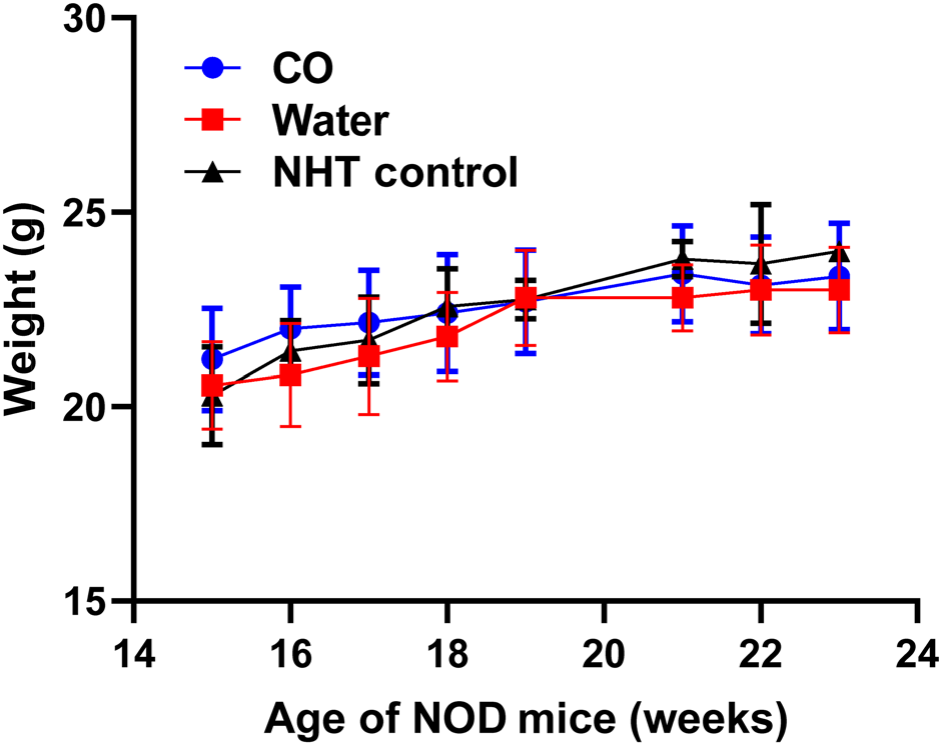
Chronic gavage delivery of CO does not impair weight gain in NOD mice. Body weights were measured weekly or biweekly starting at 15 weeks of age. Experimental groups consisted of CO‐treated (CO, *n* = 10), water‐treated (WT, *n* = 10), and no handling or treatment control group (NHT, *n* = 7). All data are expressed as mean ± SD.

Chronic oral gavage delivery of CO into the NOD model has not been documented before, and therefore, we wanted to ensure that neither the constant gavaging nor the CO extract in of itself, had any detrimental effects on the mice by way of food consumption or weight gain. Therefore, we measured the body weights of the NOD mice weekly starting at 15 weeks up until 25 weeks. NOD body weights among treatment groups were not statistically significant (*P* > 0.05, ANOVA) (Fig. 1). No harmful effects were observed in the consistently gavaged groups (CO and WT) and body weights did not differ across experimental groups including the NHT group, suggesting that oral gavage delivery of CO did not impair weight gain and potentially food consumption.

### Oral delivery of CO decreases incidence of T1D, glucose concentration and hyperglycemia in NOD mice

**Fig. 2:**
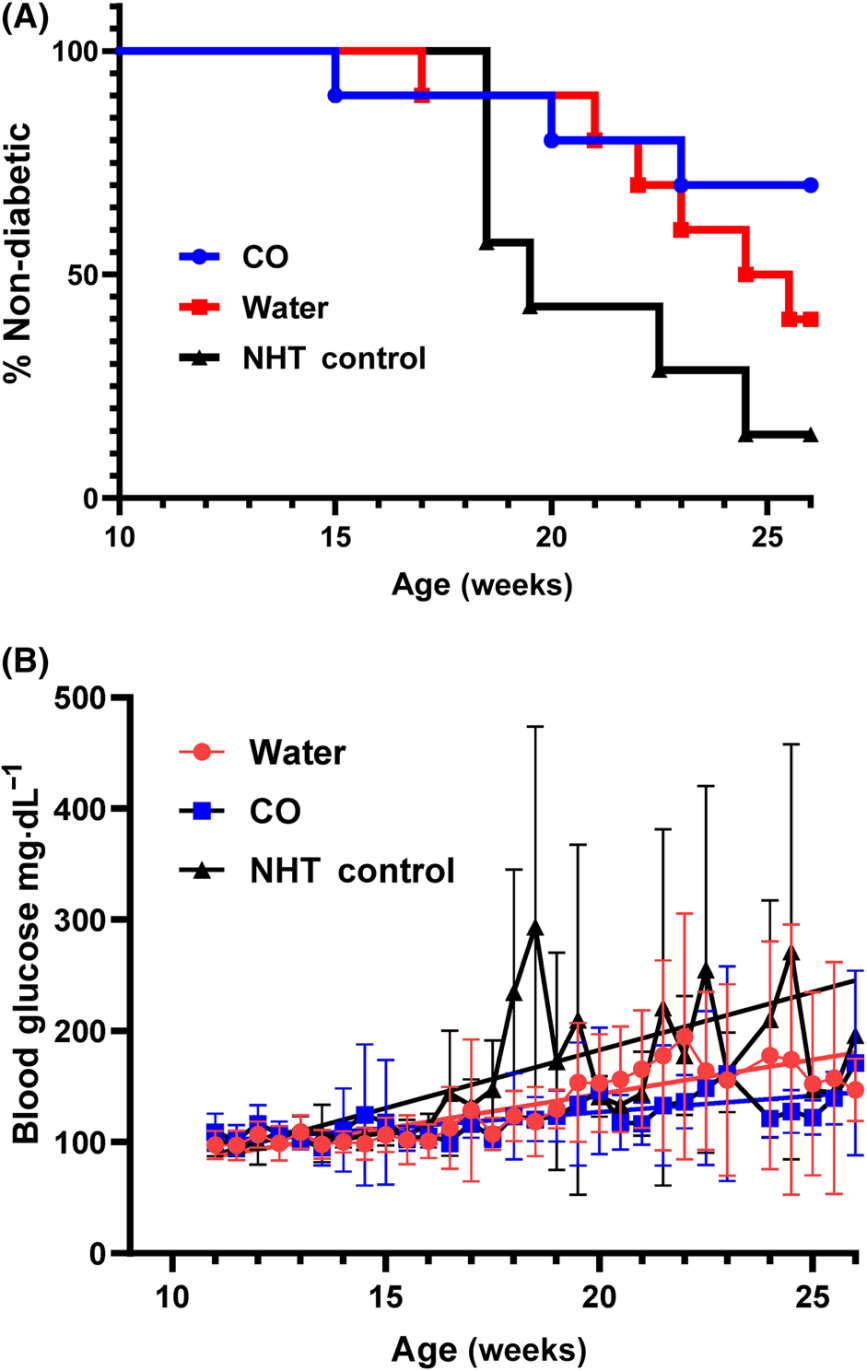
Oral gavage delivery of CO delays T1D onset and lowers average glucose concentration in NOD mice. Glucose concentration was determined from tail vein collection by commercial glucometer (Contour Next). (A) Diabetic onset was defined as two consecutive readings above 250 mg·dL^−1^. Vertical axis shows total percentage of non‐diabetic mice. Initial experimental groups consisted of CO‐treated (CO, *n* = 10), water‐treated (WT, *n* = 10), and no handling or treatment control group (NHT, *n* = 7). Log‐rank test for trend was significant with *P* < 0.05. (B) Average glucose concentration ± SD is shown from all surviving NOD mice from each treatment group displayed by age. Glucose concentration was measured following 4 h fast during AM collection. Line of best fit as determined by linear regression is shown. The glucose concentration slopes were shown to be significantly different with *P* < 0.0001.

**Fig. 3:**
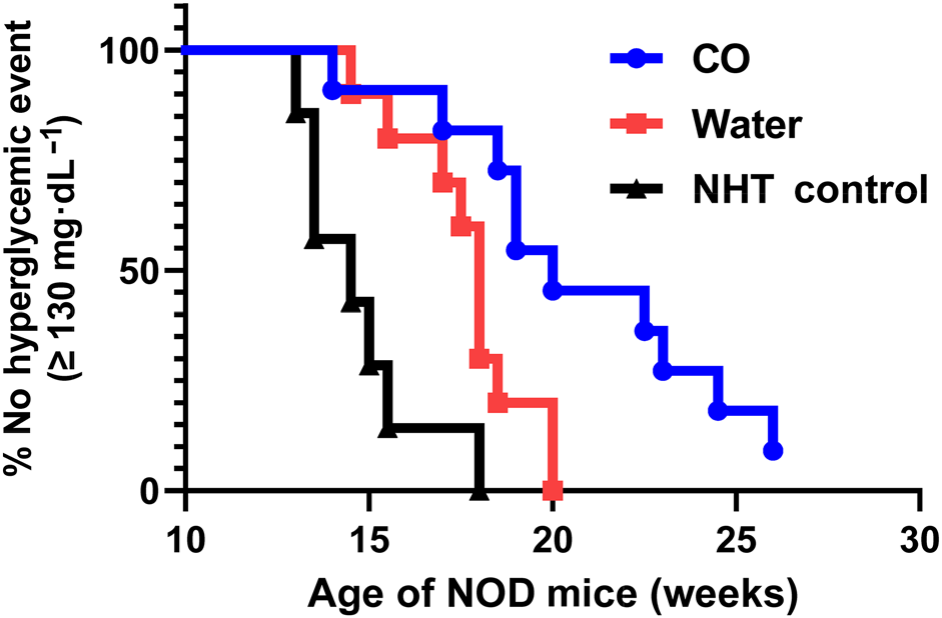
Oral gavage delivery of CO inhibits hyperglycemia in NOD mice. Hyperglycemia was defined by a single glucose measurement > 130 mg·dL^−1^ following 4 h fast as determined from tail vein collected plasma. The vertical axis shows percentage of mice without at least one hyperglycemic reading. Log‐rank test for trend was significant with *P* < 0.001.

To determine if CO inhibited the onset of T1D or hyperglycemia in the NOD mouse, we measured the blood glucose via tail vein collection approximately every 3–4 days following a 4 h fast. Diabetic onset was determined by two separate daily independent glucose readings ≥ 250 mg·mL^−1^. T1D incidence per group at 26 weeks was as follows: CO (30%), WT (60%), and NTH (86%) (Fig. 2A). The NHT control group had the highest incidence of T1D with 6 out of the 7 mice having diabetic onset by 26 weeks of age. In addition, we observed T1D onset of approximately 50% by 19 weeks of age in the NHT group as has been previously reported for the NOD model. Regarding the treatment groups, the diabetic onset between the WT and CO‐treated groups was significantly different, with the CO‐treated group showing a significantly higher proportion of non‐diabetic mice (Log rank test, *P* = 0.018) (Fig. 2A). The average glucose concentration ± SD are also shown from all NOD mice across treatment groups and displayed by age (Fig. 2B). The glucose concentration slopes were shown to be significantly different (*P* < 0.0001) across treatment groups. In addition, appearance of hyperglycemia (single glucose reading > 130 mg·dL^−1^ considered hyperglycemic event) was significantly delayed in the CO‐treated NOD mice as compared to WT and NTH groups (*P* < 0.001) (Fig. 3). By 20 weeks of age, all mice in the WT group exhibited at least one reading of hyperglycemia, whereas the observation was not matched in the CO‐treated group until 26 weeks of age.

### CO reduces insulitis in the NOD mouse

**Fig. 4:**
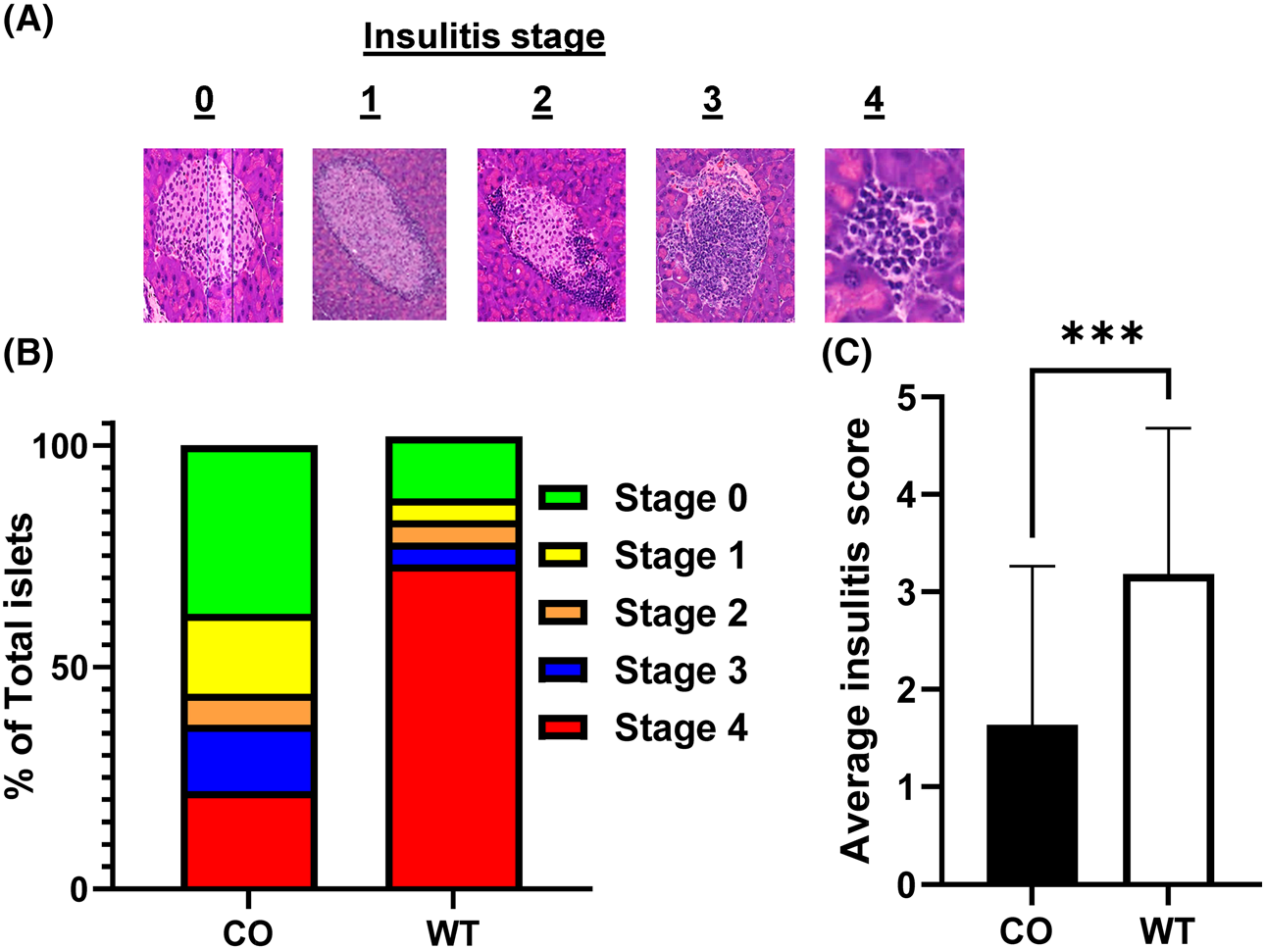
Decreased insulitis in CO‐treated NOD mice aged 26 weeks. (A) Representative images of H&E‐stained pancreatic sections obtained from CO‐treated NOD mice showing insulitis staging. (B) Insulitis stage percentage shown by insulitis category from all counted islets. (C) Average insulitis score of islets from both the CO and WT groups. All data expressed as mean ± SD. *** denotes *P* < 0.001 for average insulitis score between the CO and WT groups as determined by unpaired *t*‐test. Surviving non‐T1D NOD mice at 26 weeks of age from all treatment groups (CO; *n* = 7, WT; *n* = 4, and NHT; *n* = 1).

Insulitis is characterized as the influx of immune cells surrounding and within the pancreatic islets and is considered a distinguishing clinical characteristic of T1D and also mirrored in the NOD model [24]. Therefore, we examined insulitis in the endocrine pancreas of the surviving non‐T1D NOD mice at 26 weeks of age from all treatment groups (CO; *n* = 7, WT; *n* = 4, and NHT; *n* = 1) by performing H&E staining of pancreatic sections obtained from surviving mice at the conclusion of the study at 26 weeks. This was performed in collaboration with the University of Florida Molecular Pathology Core. Degree of insulitis was scored by staging criterion ranging from: Stage 1: Only perinsulitis observed, Stage 2: < 50% insulitis, Stage 3: > 50% insulitis, Stage 4: 100% insulitis for the pancreatic sections from the CO and WT group but not from NHT since only 1 was assessed. Representative images observed from our experiments are shown in Fig. 4A. Next, we measured the insulitis from all islets counted from each pancreatic section and expressed the insulitis staging as a percentage of all islets counted within a treatment group. In the WT group, most of the examined islets exhibited severe insulitis (73%), while the CO‐treated group had mild to no insulitis (56% stages 0 or 1) (Fig. 4B). The mean insulitis score in the CO‐treated group (*n* = 58 islets counted) was significantly lower when compared to the control WT group (*n* = 26 islets) (1.83 ± 0.22 vs. 3.12 ± 0.32, respectively, *P* < 0.001, unpaired *t*‐test) (Fig. 4C). In addition, we also performed immunophenotyping of the observed pancreatic insulitis via IHC analysis staining for CD45, CD3 and B220 serving as markers of lymphocytes, T‐cells and B‐cells respectively. No difference was observed among the treatment groups in terms of appearance and distribution regarding the observed immunophenotyping (Fig. S1). Differences in circulating levels of cytokines were also measured across surviving mice of completed regimen across treatments groups. Plasma was collected during the terminal collection and the concentration of TNF‐α and CXCL10 was measured via multiplexing analysis for CO‐ and WT‐treated groups. No significant differences were observed in circulating levels of both TNF‐α and CXCL10 between both groups (Fig. S2).

### Increased circulating C‐peptide in non‐diabetic CO‐treated mice

**Fig. 5:**
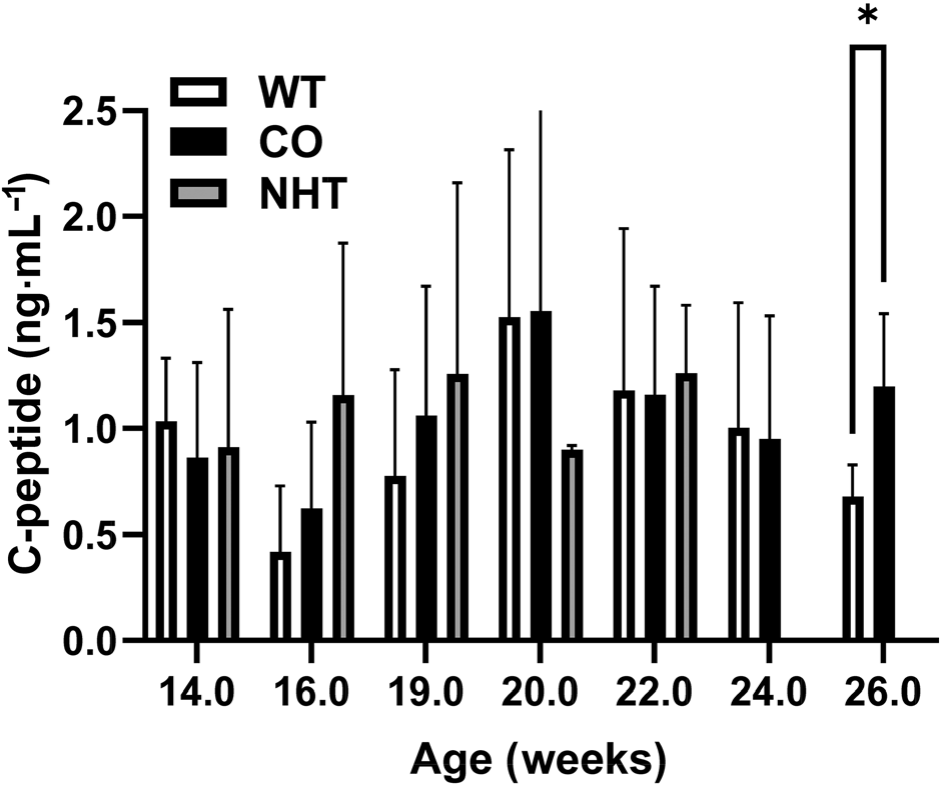
C‐peptide levels measured from CO‐treated NOD mice. Tail vein plasma was collected from all surviving mice approximately every 3 weeks. Initial experimental groups consisted of CO‐treated (CO, *n* = 10), water‐treated (WT, *n* = 10), and no handling or treatment control group (NHT, *n* = 7). C‐peptide concentration was determined by commercial ELISA (Crystal Chem). Circulating average C‐peptide levels evaluated from plasma are shown as mean ± SD. *P < 0.05 as determined by unpaired *t*‐test. C‐peptide concentration is not shown for NHT group at 24 and 26 weeks due to insufficient *N* size remaining. WT, water‐treated; CO, *Cornus officinalis*‐treated; and NHT, no treatment or handling control.

C‐peptide is secreted along with insulin by pancreatic β‐cells following enzymatic cleavage of the prohormone proinsulin to produce insulin and C‐peptide in equimolar amounts [25]. C‐peptide also more accurately represents portal insulin secretion rather than measurement of peripheral insulin [26] due to relatively fast peripheral clearance and first pass metabolism of insulin by the liver [27, 28]. As a result, C‐peptide is commonly used as a measurement for insulin secretion and pancreatic β‐cell function which is a direct measure of β‐cell viability. Therefore, we wanted to examine if CO promotes β‐cell preservation. For the reasons stated above we measured circulating C‐peptide concentration typically every 2 weeks on the surviving mice from the ages of 14–26 weeks (Fig. 5). The overall C‐peptide concentration across treatment groups longitudinally did not significantly differ until the terminal collection. At week 26, there was a significant difference in CO concentration between the suriving mice of the CO and WT groups. There was a statistically significant increase in C‐peptide concentration in the CO as compared to the WT group in the surviving mice at the conclusion of the study (1.2 ± 0.34 SD vs 0.68 ± 0.15 ng·mL^−1^, *P* < 0.05 as determined by unpaired *t*‐test, respectively).

## Discussion

*Cornus officinalis* has been shown to have several beneficial effects in pancreatic β‐cells including promotion of viability and metabolic activity in addition to cellular rescue from cytokine induced cell death [11]. Furthermore, our earlier findings demonstrated that CO could induce the Keap1/Nrf2 pathway resulting in increased expression of the critical antioxidant genes SOD2 and HO‐1 in a human pancreatic β‐cell line [12]. Since Nrf2 signaling has been shown to also repress the onset of T1D in the NOD mouse [17], we hypothesized that CO may promote these beneficial effects in the NOD model with potential translational value as an additive interventional therapy for T1D by inhibiting the progression of pancreatic β‐cell destruction.

Through this study, we showed that CO extract delivered by oral gavage delays the onset of T1D in a greater percentage of NOD mice as compared to control groups WT and NHT. Our study utilized a novel approach employing oral gavage delivery of CO. Our recent literature evaluation examining oral gavage therapy of CO has identified only 4 manuscripts reporting this approach of CO delivery and to our knowledge, none have ever examined the impact of oral gavage therapy of CO in the NOD model demonstrating the novelty of our approach in a T1D application [29, 30, 31, 32]. Our results showed that chronic gavaging of CO had no effect on the weight gain of the mice which provides some evidence that this approach is not detrimental to the overall health of the mice (Fig. 1). We assume that there were no differences in food consumption, but this was not exactly measured, and the limited conclusion is based overall similar linearity of weight again among all experimental groups especially that of the NHT control. Moreover, we showed that CO not only significantly reduced the T1D incidence (Fig. 2) at 26 weeks but we also observed a delayed appearance of hyperglycemia (Fig. 3). In addition, we also examined the degree of insulitis in the excised pancreata from the NOD mice at the conclusion of the study from surviving mice which revealed reduced severity of immune infiltration in the NOD mice associated with CO treatment (Fig. 4). Taken together, these results do suggest that there was a preservation of β‐cell function that is further supported by the significantly higher concentration of circulating C‐peptide measured in CO‐treated NOD mice at the terminal time point of surviving mice (Fig. 5). Our combined findings provide initial support of CO as a potential therapeutic agent that is safe and effective in the interventional treatment for stages 1 and 2 T1D. Stages 1 and 2 of T1D are characterized by the presence of autoimmunity against the pancreatic β‐cell without any of the typical symptoms of T1D (Ketoacidosis, weight loss, fatigue etc) while stage 2 is differentiated from stage 1 by the presence of dysglycemia [33]. Despite the appearance of autoimmunity and dysglycemia during Stages 1 and 2 T1D, there is still sufficient and functional pancreatic β‐cell mass to prevent the necessity for insulin treatment. Therefore, therapies employed during these stages such as CO may be potentially implemented as a preventative and safe measure that can inhibit and delay T1D onset.

Currently, the only FDA‐approved interventional therapy for T1D is an immunotherapy called teplizumab. Teplizumab is an anti CD3 antibody which targets the autoimmune response [5] but does not do much to restore beta cell function itself. Considering that β‐cell stress has been shown to precede insulitis [34], trying to maintain β‐cell homeostasis is crucial. β‐Cell homeostasis is so critical in fact that current research has shown that β‐cell stress can result in the creation of “Neo‐antigens” and the onset of autoimmunity [35]. For the reasons stated above, it is important to identify interventional treatments that not only prevent autoimmunity but also preserve β‐cell function. CO may be beneficial in identifying further compounds which may help maintain β‐cell homeostasis and could provide an avenue for new interventional therapies.

Even though we showed the potential therapeutic utility of CO for T1D preventative therapy there are still limitations that need to be addressed. Although the chronic oral gavage delivery did not appear to have a detrimental effect there was a substantial delay in T1D onset between the no handling control and the WT control. As our data demonstrated, by 19 weeks our NHT group displayed T1D onset of 50%, whereas the WT group did not reach this level until 24 weeks. This “handling effect” resulted in an overall delay in T1D onset for oral gavaged WT group. The precise reason for this effect is not exactly known but we hypothesize that the chronic stress of handling induces an immunosuppressive effect resulting in the delayed T1D onset. Going forward, it will be necessary to devise a method for CO delivery that is not as invasive as oral gavaging and importantly not inducing an overall T1D delay in the NOD model.

Another critical limitation of our findings is that this reported study has not addressed the precise mechanism of how CO may be inhibiting T1D onset or hyperglycemia. CO has been reported to have numerous pleiotropic effects on both metabolism and inflammation [36]. CO has been shown to promote glucose sensitivity through mechanisms such as promoting hepatic glucose uptake as well as anti‐inflammatory effects via suppression of phosphorylation of STAT3 and NF‐κB. Whether the metabolic or anti‐inflammatory effects alone or both in combination explains in part our results have yet to be fully evaluated and are beyond the scope of this current study. However, this report was the critical first step to determine if there is biological efficacy of CO in a widely supported and tested model of T1D. Future studies examining the impact of CO on T1D should incorporate additional immunological markers such as examination of T‐cell subsets, generation of Regulatory T cells (Tregs), and the impact on lymphocyte trafficking. Other studies have demonstrated that administration of CO in animal models can alter the intestinal microbiome and promote various metabolic effects [37, 38]. Therefore further examination should be performed elucidating any changes in the gut microbiome during CO administration.

Our earlier analysis has revealed that CO is able to promote a robust antioxidant response by increasing expression of SOD‐2 and HO‐1 along with stimulating autophagy. We will need to confirm that this pathway is responsible for the T1D delay observed in the NOD mouse. Moreover, CO is a complex mixture of many different compounds and trying to identify individual compounds which are responsible for the observed biological effects such as delaying T1D will be crucial for elucidating direct agonist actions of CO involve and the potential transfer to therapeutic usage.

In summary, the extract from CO significantly delayed the onset of T1D in the NOD mouse. Even though further research is needed to confirm the mechanism of action, CO may be preserving β‐cell function by promoting β‐cell viability thus making CO a promising source for new potential interventional therapies.

## Conflict of interest

The authors declare no conflict of interest.

### Peer review

The peer review history for this article is available at https://www.webofscience.com/api/gateway/wos/peer‐review/10.1002/2211‐5463.13758.

## Author contributions

JDF and BRB conceived and supervised the study; JDF and GEO performed experiments; YCZ provided and advised on the preparation of the *C. officinalis*; JDF, GEO and BRB analyzed data; JDF, GEO and BRB wrote the manuscript; JDF and BRB made manuscript revisions.

## Supporting information


**Fig. S1.** Immunophenotyping of pancreatic endocrine insulitis. IHC staining was performed on pancreatic sections obtained from remaining mice following 15 treatment weeks. IHC analysis staining for CD45, CD3 and B220 serving as markers of lymphocytes, T‐cells, and B‐cells respectively were utilized for evaluation of immune populations present in the endocrine pancreas from the CO, WT, and NHT groups. Representative images are shown.

**Fig. S2.** Measurement of circulating TNF‐α and CXCL10 via multiplexing analysis. Plasma was collected from the surviving mice from the CO (*n* = 7), WT (*n* = 4) and NHT (*n* = 1) treatment groups following 15 treatment weeks and examined for the concentration of TNF‐α and CXCL10 via multiplexing analysis. Measurements are shown as mean ± SD. *P* > 0.05 as determined by unpaired *t*‐test.

## Data Availability

The data that support the findings of this study are available from the corresponding author upon reasonable request.
