# Supplementary figures and images for: Oral gavage delivery of Cornus officinalis extract delays type 1 diabetes onset and hyperglycemia in non‐obese diabetic (NOD) mice
Source: FEBS Open Bio. 2024 Jan 9;14(3):434–43. doi: 10.1002/2211-5463.13758 (PMC10909980; doi:10.1002/2211-5463.13758)

Supplemental Figure 1. Fletcher et al.

**CD45**

**CD3**

**B220**


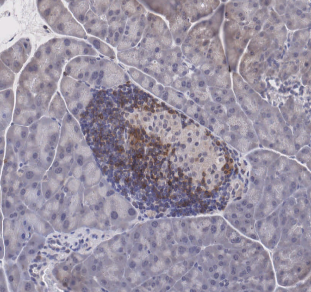

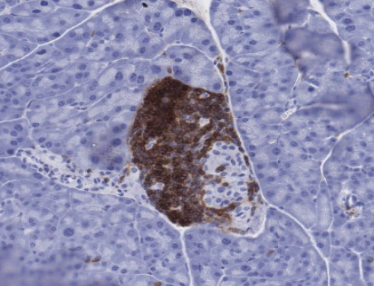

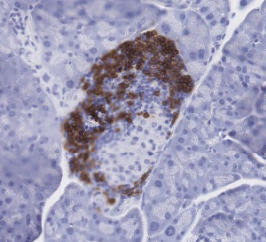

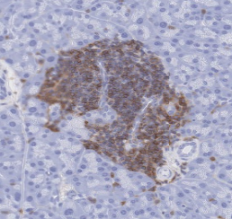

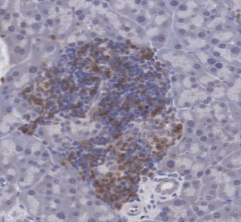

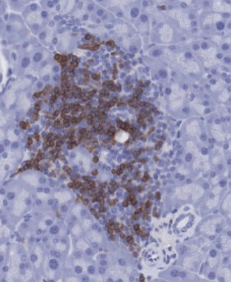

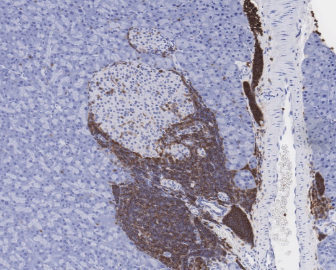

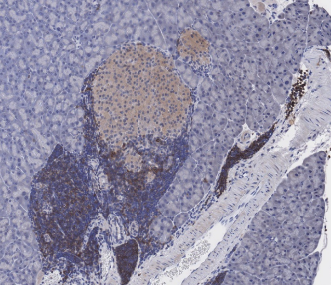

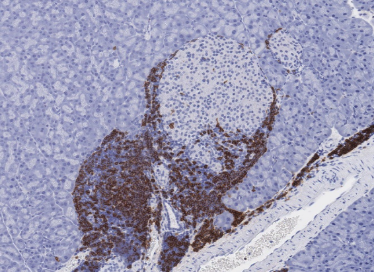


**CO**

**NHT**

**WT**

Supplement: Supplementary file 1 — Fig. S1. Immunophenotyping of pancreatic endocrine insulitis. IHC staining was performed on pancreatic sections obtained from remaining mice following 15 treatment weeks. IHC analysis staining for CD45, CD3 and B220 serving as markers of lymphocytes, T‐cells, and B‐cells respectively were utilized for evaluation of immune populations present in the endocrine pancreas from the CO, WT, and NHT groups. Representative images are shown. [file FEB4-14-434-s001.docx]
