# Supplementary material for: Oral gavage delivery of Cornus officinalis extract delays type 1 diabetes onset and hyperglycemia in non‐obese diabetic (NOD) mice
Source: FEBS Open Bio. 2024 Jan 9;14(3):434–43. doi: 10.1002/2211-5463.13758 (PMC10909980; doi:10.1002/2211-5463.13758)
Supplement: Supplementary file 2 — Fig. S2. Measurement of circulating TNF‐α and CXCL10 via multiplexing analysis. Plasma was collected from the surviving mice from the CO (n = 7), WT (n = 4) and NHT (n = 1) treatment groups following 15 treatment weeks and examined for the concentration of TNF‐α and CXCL10 via multiplexing analysis. Measurements are shown as mean ± SD. P > 0.05 as determined by unpaired t‐test. [file FEB4-14-434-s002.docx]

Supplemental Figure 2. Fletcher et al.
